# Cardiac mechanics and reverse remodelling under mechanical support from left ventricular assist devices

**DOI:** 10.3389/fcvm.2023.1212875

**Published:** 2023-08-02

**Authors:** Blanca Pamias-Lopez, Michael E. Ibrahim, Fotios G. Pitoulis

**Affiliations:** ^1^ Department of Myocardial Function, Imperial College London, National Heart and Lung Institute, London, United Kingdom; ^2^ Division of Cardiovascular Surgery, University of Pennsylvania School of Medicine, Philadelphia, PA, United States

**Keywords:** cardiac remodeling, left ventricular assist device (LVAD), impella, pressure volume (PV) curve, reverse remodeling, hemodynamics

## Abstract

In recent years, development of mechanical circulatory support devices has proved to be a new treatment modality, in addition to standard pharmacological therapy, for patients with heart failure or acutely depressed cardiac function. These include left ventricular assist devices, which mechanically unload the heart when implanted. As a result, they profoundly affect the acute cardiac mechanics, which in turn, carry long-term consequences on myocardial function and structural function. Multiple studies have shown that, when implanted, mechanical circulatory assist devices lead to reverse remodelling, a process whereby the diseased myocardium reverts to a healthier-like state. Here, we start by first providing the reader with an overview of cardiac mechanics and important hemodynamic parameters. We then introduce left ventricular assist devices and describe their mode of operation as well as their impact on the hemodynamics. Changes in cardiac mechanics caused by device implantation are then extrapolated in time, and the long-term consequences on myocardial phenotype, as well as the physiological basis for these, is investigated.

## Introduction

Chronic pathological hemodynamic stress following an index event (e.g., pressure-, or-volume-overload) alters myocardial molecular, cellular, and interstitial factors, manifesting clinically as changes in size, shape, and function of the heart. Each stressor can affect the operation and mechanics of the heart distinctly across time and with often different underlying mechanisms (1, 2). However, when left uncorrected prolonged hemodynamic overload ubiquitously leads to heart failure (HF), characterised by dilation of heart size and deterioration of contractile function. The development of mechanical support devices has provided a new way to both halt the vicious cycle of haemodynamic overload before progression to HF, but also to support the failing heart, allowing reversal of overload-induced structural and functional pathology, in a process termed reverse remodelling.

Mechanical support devices can provide univentricular or biventricular support, can be implanted percutaneously or surgically, provide varying amounts of cardiac output (CO) support and for different durations (3). An important distinction is that although all mechanical support devices provide some degree of circulatory support, not all circulatory support devices mechanically unload the myocardium, and some may even overload it. For example, veno-arterial extracorporeal membrane oxygenation (VA-ECMO) is becoming an established modality to provide circulatory support in the setting of cardiogenic shock, yet it retrogradely perfuses the aorta severely increasing left ventricular (LV) afterload and overloading the myocardium (4, 5).

In this review we focus exclusively on LV assist devices (LVADs)—that is, those that mechanically unload the LV. These include both durable LVADs (d-LVADs), such as the HeartMate 3, which are surgically implanted and can be used for prolonged periods of time, as well as transvalvular micro-axial flow pump devices (tma-LVAD), such as the Impella, which can be percutaneously implanted and are typically used for acute mechanical support and for shorter durations (6). We begin by examining the mechanics of healthy myocardium, assessed in the pressure-volume (PV) plane, and the expected changes to these with progression to HF. Subsequently, we investigate how LVADs alter cardiac mechanics, immediately upon implantation but also in the long-term and how changes in mechanical load may lead to reverse remodelling, as well as the structural and functional changes and implicated underlying mechanisms of this.

### Pressure-volume relationships

To understand the effects of mechanical support devices on cardiac mechanics an understanding of pressure-volume (PV) loops is required. A brief description of the main determinants of cardiac mechanics is shown here—for a more detailed review see (7).

**Figure 1:**
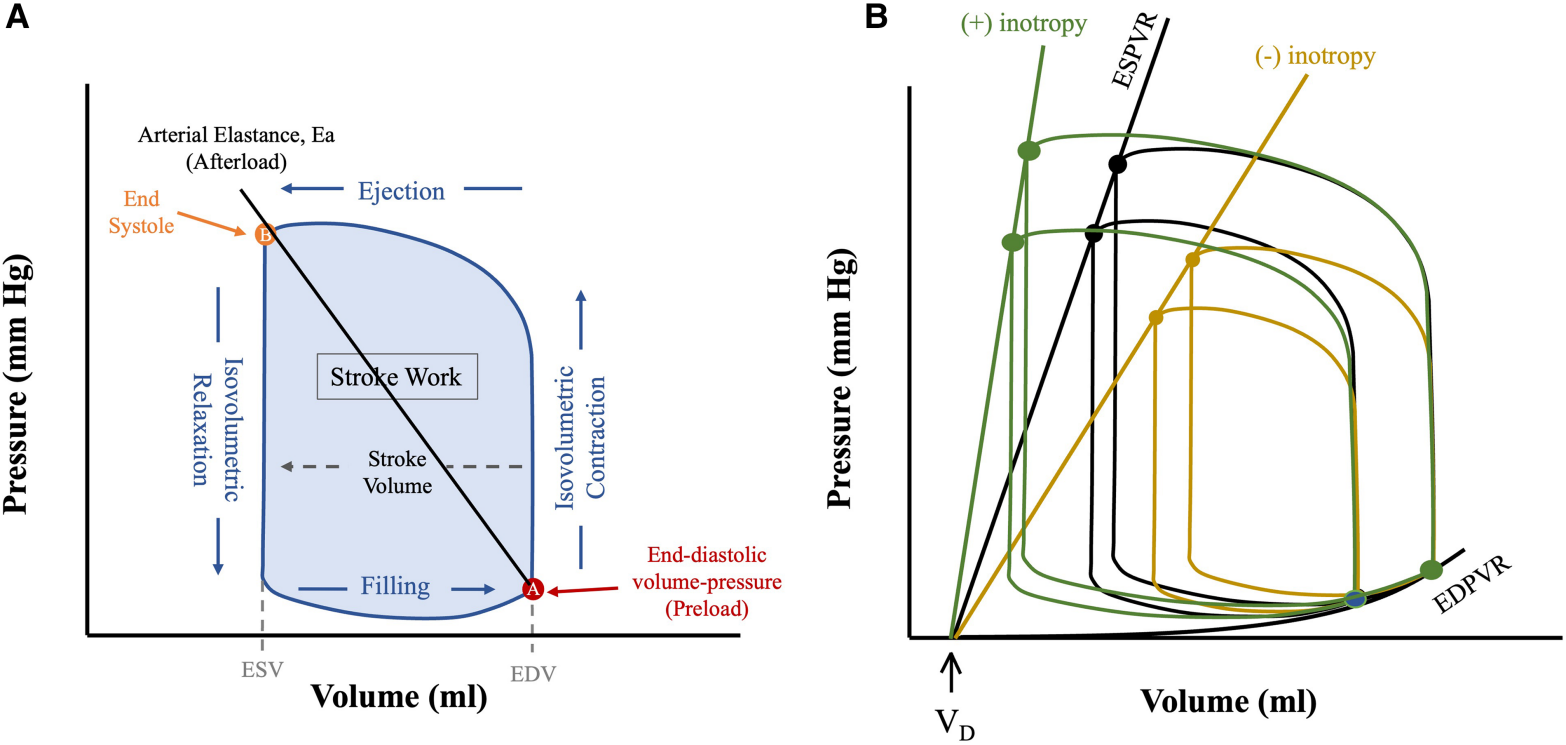
Anatomy of a PV loop. (**A**) Normal PV loop representing the 4 phases of the cardiac cycle, as the heart transitions from diastolic (**A**) to systolic (**B**) state and back. (**B**) Pressure-volume loops at different preloads and contractile states (*E*_es_). All PV loops are bounded by their corresponding linear ESPVR (drawn from the instantaneous end-systolic pressure-volume points) and the EDPVR (drawn from the corresponding end-diastolic pressure-volume points). The slope of ESPVR represents end-systolic elastance (*E*_es_), a measure of intrinsic contractility, and the *x*-axis intercept is the dead volume (*V*_d_), below which ventricular pressure generation does not occur. Figures are adapted from: (8). ESV, end systolic volume; EDV, end diastolic volume.

Cardiac mechanical load is characterized by afterload and preload, both of which can alter stroke volume (SV) via the Frank-Starling relationship. Preload refers to the mechanical load the heart is exposed to prior to the beginning of systole—it is appreciated by the end-diastolic pressure or volume point in the PV loop (Figure 1). Afterload refers to the mechanical load against which the ventricle contracts and can generally be appreciated by the arterial elastance (*E*_a_) shown as a line that connects the end-diastolic (EDV) and end-systolic (ESV) volume points (Figure 1). The Frank-Starling mechanism relates preload to SV, whereby increased preload results in increased SV.

The PV loop is demarcated by the 4 phases of the cardiac cycle (Figure 1): (1) isovolumetric contraction, (2) ejection, (3) isovolumetric relaxation and (4) filling. The width denotes SV, and the area inside the PV loop represents stroke work, which correlates to myocardial oxygen consumption (MVO_2_) (Figure 1) (9) (*see below*). Cardiac function is dependent upon preload and afterload and so within the *in vivo* state where these are constantly varying it is challenging to assess unless they are well controlled for (10) developed a mathematical function, termed time-varying elastance, *E*(*t*), that has enabled determination of cardiac function irrespective of mechanical load. *E*(*t*) is a load-independent measure of contractile state and is defined using Equation (1) as shown below:

(1) $$E(t)=\frac{P(t)}{V(t)-V_{D}}$$

**Figure 2:**
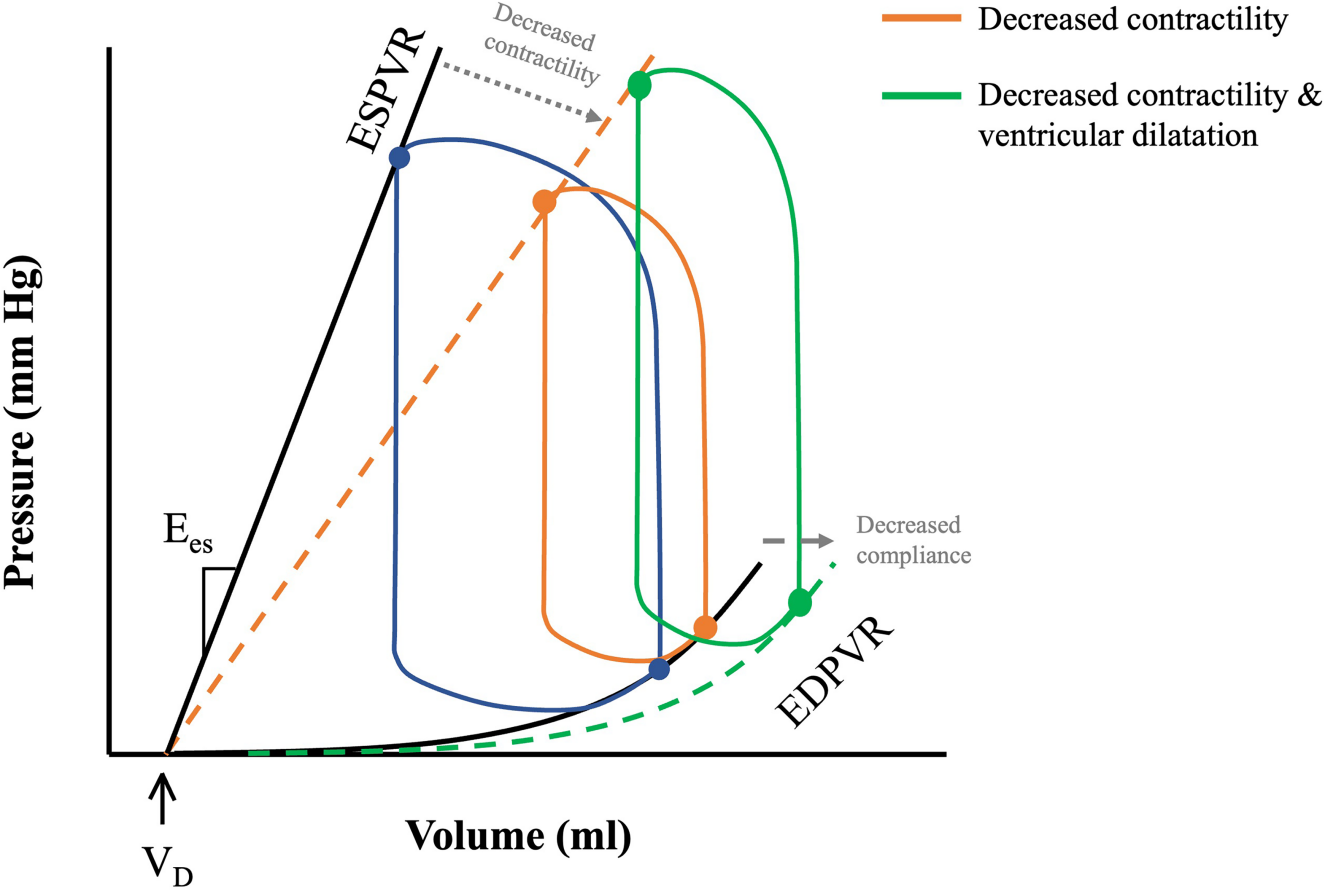
PV loops in health and disease. ESPVR and EDVPR shifts with decreasing contractility and decreased compliance (depicted by the grey dotted arrows) during pathological remodelling of the heart. Decreased cardiac contractility in HFrEF shifts ESPVR rightwards with a shallower slope (orange dotted line). Progressive ventricular remodelling leads to a rightward shift of the EDPVR (green dotted line), and further downward shifting of the ESPVR, causing LV dilatation and decreased LV contractility. Adapted from: (13).

Where *E*(*t*) is the instantaneous elastance, *P*(*t*) and *V*(*t*) instantaneous pressure and volume respectively and *V_D_* is dead volume—that is, the volume below which no pressure generation occurs in the heart (11). The maximum elastance, or end-systolic elastance (*E*_es_), can be approximated by the instantaneous end-systolic pressure-volume relationship (ESPVR), which is a load-independent measure of intrinsic contractility (Figure 1B). Increased contractility, known as positive inotropy, shifts the ESPVR leftward and up, with a steeper slope (*E*_es_), while decreased contractility, known as decreased inotropy, has opposite effect (12). A shallow ESPVR is seen in patients with a well-compensated HF with reduced ejection fraction (HFrEF) due to contractile dysfunction (Figure 2). Importantly, the shallower the ESPVR the more afterload-sensitive the heart, such that afterload-reducing interventions can have significant impact on cardiac hemodynamics and symptoms (14). The diastolic properties of the ventricle are defined by the non-linear end-diastolic pressure-volume relationship (EDPVR), which represents passive myocardial stiffness (or ventricular compliance) (15). Pathological ventricular dilatation results in a rightward shift of the EDPVR towards higher volumes, and reverse remodelling following mechanical unloading has been shown to shift it back towards near normal levels (16). Both ESPVR and EDPVR can be used to evaluate myocardial tissue properties in progression from healthy to failing myocardium and vice versa, irrespective of loading conditions (11, 14).

## Mechanical circulatory support devices

Mechanical support devices are often used clinically in the management of patients with end-stage HF and acute hemodynamic compromise. Although there are many different types of devices and indications for use, in this paper we discuss d-LVADs and trans-axial tma-LVADs. The mode of operation of these is generally the same, namely, to unload the heart by extracting blood from the LV and ejecting it into the aorta, however, they differ in terms of implantation method, indications for use, and duration of treatment.

### Durable left ventricular assist devices

**Figure 3:**
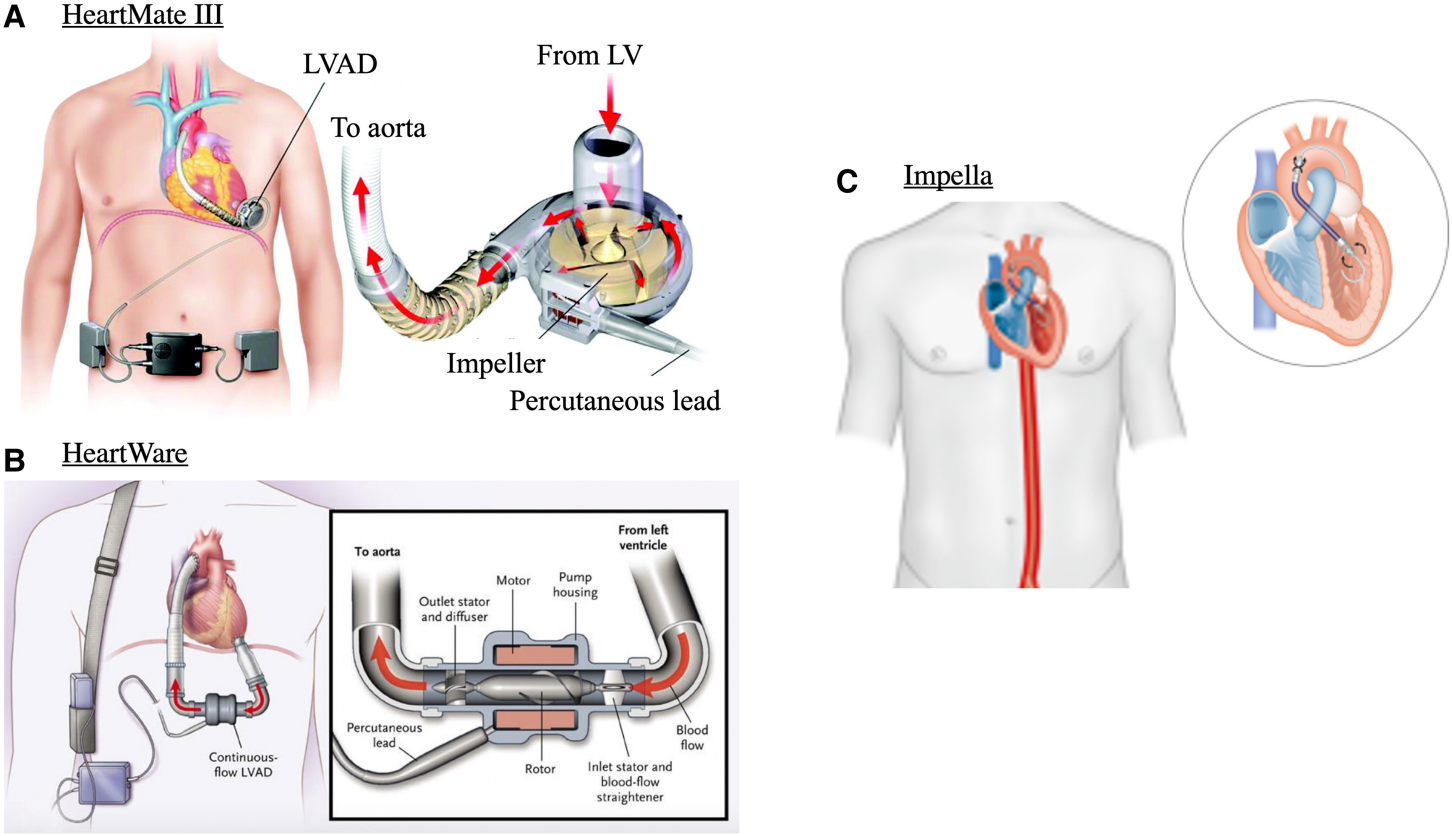
Mechanical circulatory support devices. (**A**) centrifugal continuous flow durable LVAD (HeartMate 3), (**B**) continuous axial-flow durable LVAD (HeartWare) and (**C**) Transvalvular micro-axial flow device (Impella). Images adapted from: (17–19).

d-LVADs refer to fully implantable devices that provide circulatory support by enhancing systemic blood pressure and CO, while mechanically unloading the LV (Figure 3). The basic principle of LVADs consists of an inflow cannula in the LV apex and an outflow graft in the aorta. This design has remained unchanged despite multiple newer iterations in the operation of the device and implantation method (20).

First generation pulsatile-flow devices have been replaced by smaller, quieter, and more durable continuous-flow LVADs. These can be either centrifugal-flow (HeartMate 3, Figure 3A) or axial-flow pumps (HeartMate 2, and HeartWare, Figure 3B) (20). Continuous-flow LVADs have generally demonstrated improved end-organ function, and quality of life over pulsatile-flow devices (18, 21). This is interesting given the unphysiological nature of continuous flow and is likely related to decreased risk for thrombosis and embolic episodes due to absence of mechanical bearings in continuous flow LVADs (22). Between continuous-flow devices, centrifugal pumps carry superior outcomes to axial-flow pumps as evidenced by the 5-year outcomes of the MOMENTUM 3 randomised clinical trial. This study compared HeartMate 3 with HeartWare and demonstrated greater composite outcomes of survival to transplant, recovery, and LVAD support free from reoperation or stroke (23). d-LVADs are currently used under three main categories: bridge-to-transplantation, bridge-to-recovery, or as destination therapy (20, 24).

Bridge-to-recovery refers to sufficient myocardial recovery such that device removal is possible; this has been particularly useful in view of the limited number of donor hearts. Evidence that mechanical unloading can lead to sufficient recovery such as to enable LVAD explantation were demonstrated early on by (25). In patients with idiopathic dilated cardiomyopathy, associated with high levels of anti-β1-adrenoceptor autoantibodies (a-β1-AABs), mechanical support with LVAD led to substantial improvement in cardiac structure, function, as well as decreased a-β1-AABs titre levels such that device could be explanted (25). These early studies pioneered the protocol to evaluate for cardiac recovery, as well as developed methods to stratify patients likely to benefit from mechanical support weaning (25). For example, patients with LV internal diastolic diameter and myocardial fibrosis towards the high end of the distribution were less likely to show functional improvement (25). Likewise, to determine suitability of patients for weaning off mechanical support, repeated “off-pump” trials were used. During these, in most patients, LV ejection fraction greater than 45% and LV internal diastolic diameter less than 55 mm were considered cut-offs that signified sufficient cardiac recovery for weaning had occurred (26, 27).

Registry studies have shown that 1%–2% of patients undergo LV recovery to a degree allowing explantation (28–30). However, this low rate of recovery has been questioned as patients who are placed on mechanical support are typically those not-responding to medical therapy; their medication regimen following d-LVAD implantation is thus often poorly optimised, leading to inferior recovery rates. In fact, aggressive medical optimisation as shown in the RESTAGE-HF trial coupled with uniform LVAD protocol resulted in 40% recovery rates (31). Importantly, the findings of this trial were centre-independent suggesting an intrinsic advantage to optimised medical therapy when combined with LVAD for recovery. Recovery rates are important, as survival post-recovery is comparable to that of heart transplants (32).

Aside from paucity of donor hearts, eligibility for cardiac transplantation remains a prominent barrier to treatment of advanced HF. LVADs as destination therapy refers to permanent device support as the last resort for patients ineligible for transplantation, to prolong life while providing better quality of life. Since its FDA approval in 2010, better event-free survival, symptoms and quality of life have been demonstrated with destination therapy over optimal medical management for patients that fit specific hemodynamic profiles (33–35). Destination therapy also allows healthcare centres without transplant resources to provide treatment for this patient cohort. In fact, Brinkley et al. concluded similar outcomes following destination therapy LVAD implantation between transplant and non-transplant centres, at 1 and 12-months (36). However, it is important to note that such program success requires sufficient infrastructure for multidisciplinary team support to assist in the long-term care of these patients.

### Transvalvular micro-axial flow pump LVADs

Transvalvular micro-axial flow pumps are mechanical and circulatory support devices that when implanted sit between the LV and aortic root (Figure 3C). There are different types of tma-LVADs, most notably the Impella devices by Abiomed. These are implanted percutaneously or with surgical cut-down, and like d-LVADs continuously push blood from the LV, by means of a rotating helical screw, into the aorta (37). There are many different types of tma-LVADs, the indications and capabilities of each were recently described in detail in (37). Generally, the effects on hemodynamics are the same but depending on device, CO can be supplemented from 2.5 L/min up to 6 L/min (38, 39). As some tma-LVADs can be implanted percutaneously, deployment is faster than d-LVADs, making these devices utilisable for mechanical support in the acute setting. This allows CO and tissue perfusion to be maintained in states of hemodynamic compromise, such as cardiogenic shock. Whether tma-LVADs provide mortality benefit to these patients compared to other percutaneously implanted devices, such as the intra-aortic balloon, is still an area of active research. Though the Impella is more powerful, and thus affords greater degrees of circulatory support, data shows that it carries higher risk of complications, likely related to bigger catheter size, or need for surgical cut-down (40). As such, only patient populations that require heavy circulatory support, that cannot be managed with pharmacological support alone or other circulatory support devices (e.g., intra-aortic balloon) may be warranted to receive prognostic benefit (41).

In the following sections we look at the effects of d-LVADs and tma-LVADs on acute hemodynamic profiles, as well as the long-term consequences of these on cardiac function and structure.

### Hemodynamics under LVAD mechanical support

**Figure 4:**
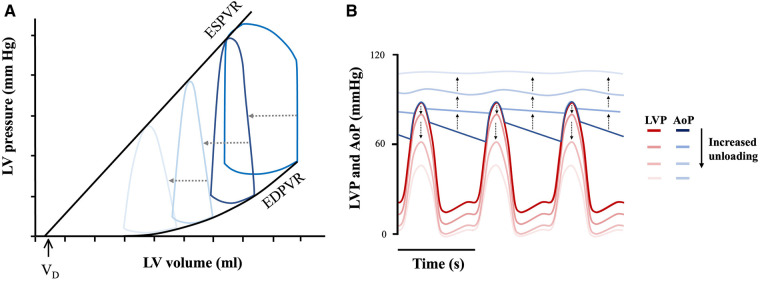
Mechanical circulatory support unloading and effect on cardiac mechanics. (**A**) Triangular PV loop with progressive leftwards and downwards shift under mechanical support and (**B**) LV-aortic pressure uncoupling. LV pressure (LVP—represented by the red theme), and aortic pressure (AoP—represented by the blue theme) progressively uncouple with increased device flow rates. Adapted from: (12, 43).

During LVAD mechanical support, blood is continually (and non-physiologically) pumped from the LV to the aorta irrespective of cardiac cycle phase. The continuous antegrade flow leads to a loss of the isovolumic contraction phase of the cardiac cycle, resulting in a “triangular” shaped PV loop that shifts down and to the left (Figure 4A) (42).

**Figure 5:**
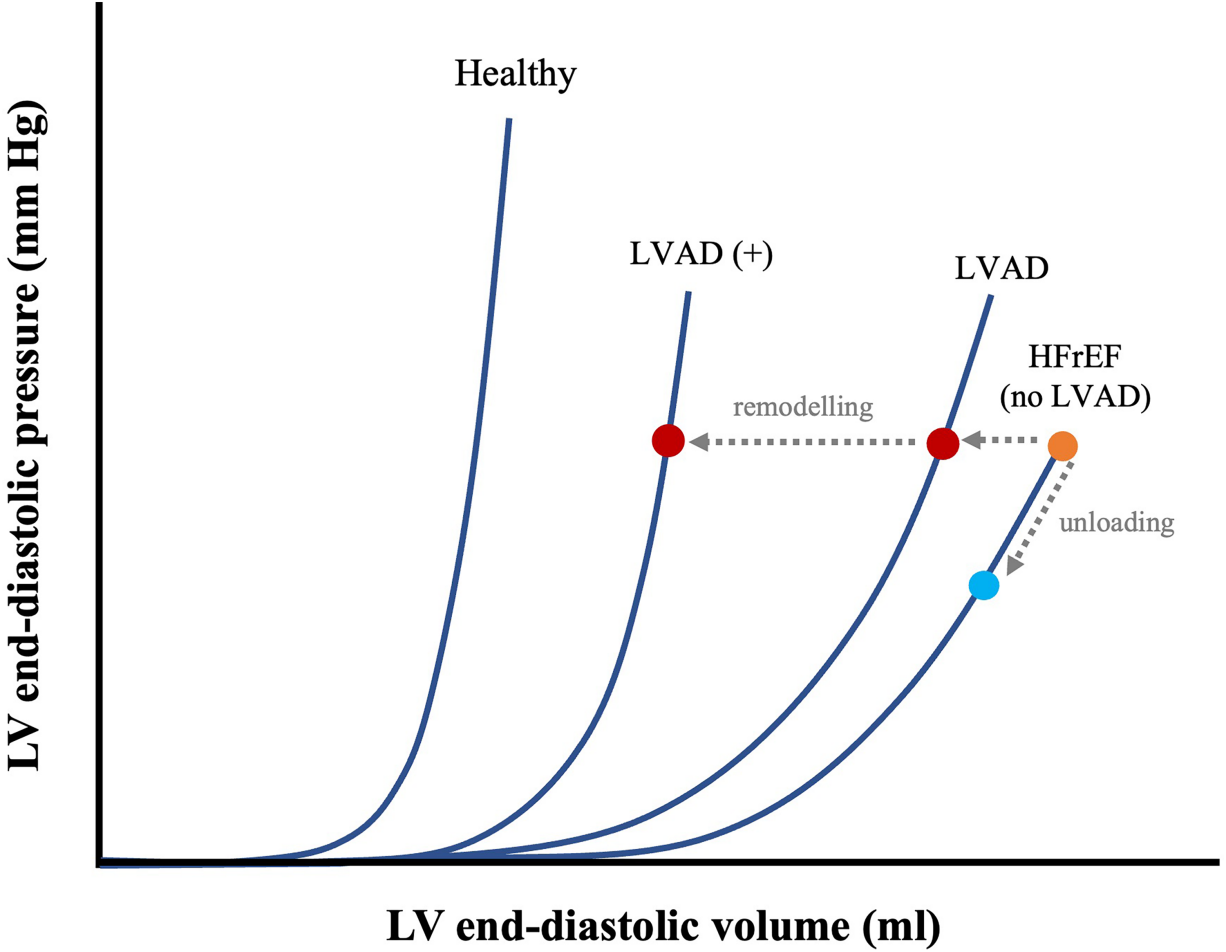
Effects of prolonged mechanical unloading on diastolic function. EDPVR of a healthy vs. HF with reduced ejection fraction (HFrEF) heart and time-dependent LVAD–associated leftward shifts in EDPVR, with shorter (LVAD) and longer duration (LVAD+) of unloading. The effects of immediate mechanical unloading on the point of operation of the heart on the EDPVR are also shown (HFrEF diagonal arrow from orange dot to blue dot). Adapted from: (43).

The degree to which the loop will shift to the left, and thus the LV will be unloaded, depends on the extent of support provided by the device (i.e., its flow-rate), which is adjustable (12). Increasing flow-rate increases the amount of blood flowing through the device into the arterial circulation, consequently supplementing CO to a greater degree. This causes a dissociation between LV pressure and aortic pressure as shown in Figure 4B (12). This flow-dependent dissociation is termed LV-aortic pressure uncoupling and it correlates quantitatively to mechanical unloading (44). Such quantifications are in turn important, as degree of mechanical support linearly correlate with myocardial remodelling (45). For example, immediately upon device implantation, flow-dependent decreases in LV end-diastolic pressure (LVEDP) are seen (44). When applied over prolonged durations, this results in a progressive leftward shift in the EDPVR, though not reaching levels seen in normal healthy hearts, as well as with decreased compliance (seen by the increased EDPVR slope) (Figure 5) (43). Thus, although not fully normalized, a lower EDPVR correlates to improved myocardial stiffness, and higher sensitivity to mechanical load (2, 46). Compared to unsupported hearts, LVADs can therefore, to a degree, reverse the negative hemodynamic effects of diastolic dysfunction (43, 46).

**Figure 6:**
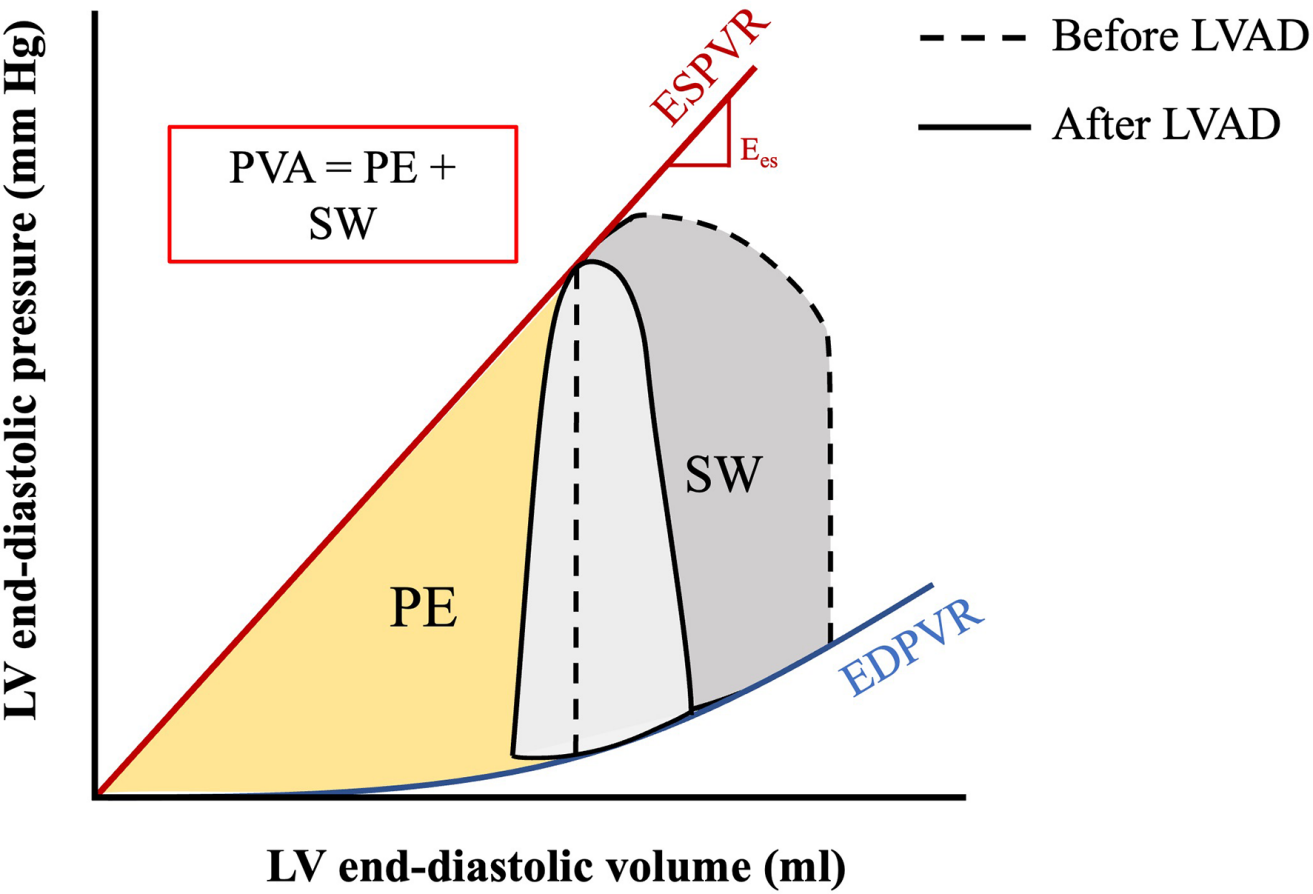
PV area corresponding to myocardial oxygen consumption. The pressure-volume area (PVA) is the sum of the stroke work (SW) and potential energy (PE) and represents the total mechanical work of the ventricle per beat. The PVA is directly correlated to the myocardial oxygen consumption (MVO_2_). Figure shows PVA before and after implantation of LVAD. Adapted from: (44).

**Figure 7:**
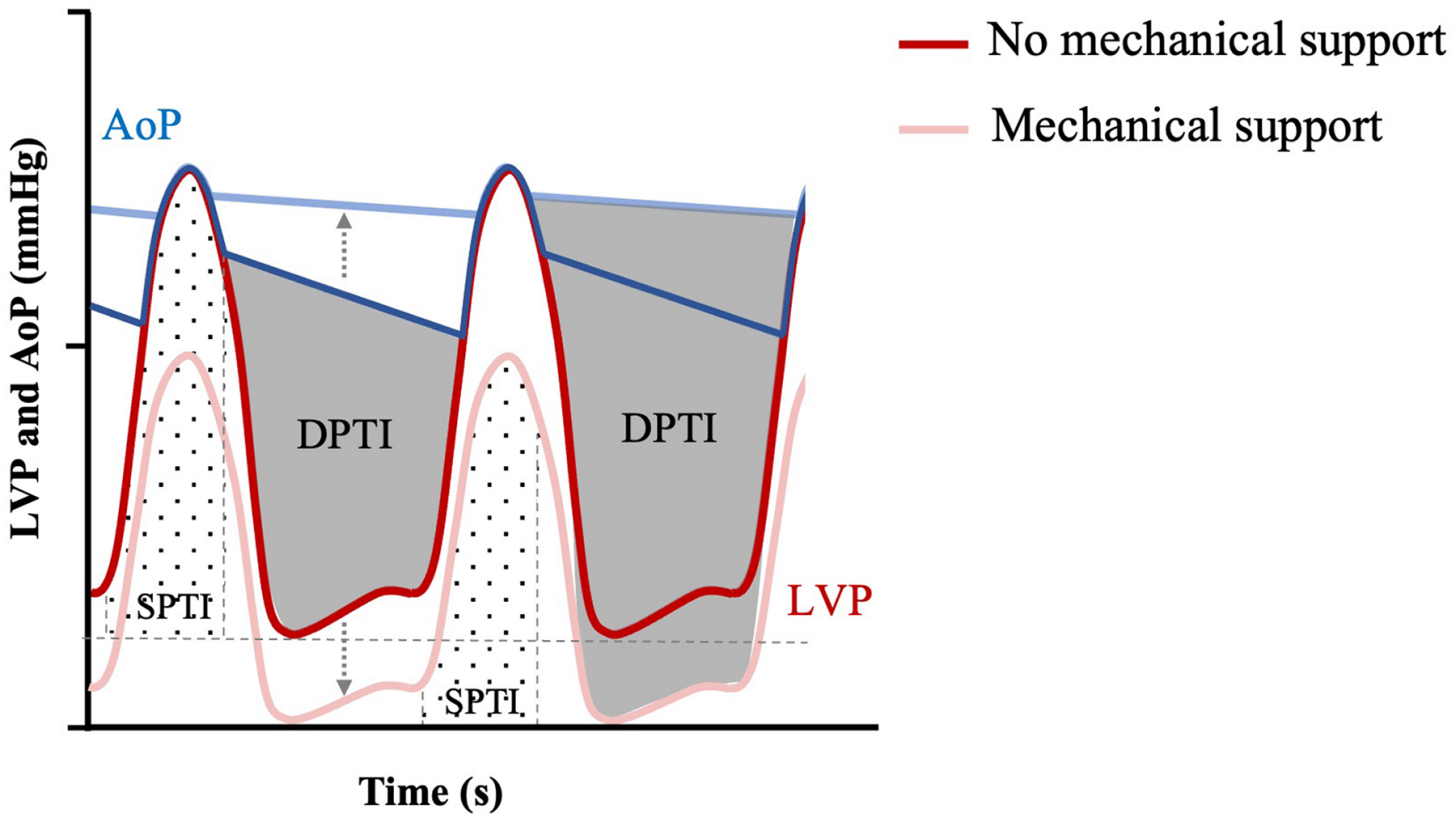
Myocardial O2 demand (SPTI) and supply (DPTI) with or without mechanical unloading. Modified Wigger's diagram showing progressive mechanical unloading (represented by grey dotted arrow) and uncoupling of LV pressure (LVP—red themed loops) and aortic pressure (AoP—blue themed loops), correlated with increased DPTI (grey overlay) and decreased SPTI (hashed overlay. Adapted from: (47).

Myocardial oxygen demand, MVO_2_, is determined by the area under the PV loop curve and corresponds to stroke work plus potential energy (Figure 6). MVO_2_ can also be appreciated in Wiggers diagrams and has been termed systolic left ventricular pressure curve index (SPTI) (47, 48) (Figure 7) (9). demonstrated a close relationship between the area under the curve of a PV loop and MVO_2_: that is—the greater the area under the curve in a PV loop or the higher the SPTI, the higher the MVO_2_. Accordingly, interventions that decrease these also decrease MVO_2_.

Under mechanical support, part of the CO is provided by the assist device. As a result, preload, and consequently (a) diastolic stress on the heart, and (b) stroke volume (given Frank-Starling) decrease. In hemodynamically unstable patients this is critical, as decreased myocardial stress correlates to decreased MVO_2_ in accordance with Laplace's law of the heart, protecting the heart from ischemic injury (49). Decreased MVO_2_ due to mechanical unloading also has profound long-term consequences. For example, implantation of Impella within 24 h in patients with anterior ST-segment myocardial infarction is associated with an increased left ventricular ejection fraction after ∼4 months from index event compared to control (50). The Protect II randomised controlled trial, showed that Impella provided greater CO power, a metric correlated to mortality (51), compared to other mechanical support devices in cardiogenic shock patients (52). Of note is that the prognostic significance of CO power may be limited to acutely decompensated patients following myocardial infarction only and not apply to patients with acute HF cardiogenic shock (53, 54). Other trials with lower-sample sizes (IMPRESS) did not corroborate these findings (55). Part of these beneficial effects may be explained by decreased infarct size in patients who received acute mechanical unloading, as has been shown in separate studies conducted in dogs and sheep that were subjected to myocardial infarcts by left anterior descending coronary artery ligation followed by mechanical unloading (45, 56).

In addition to lowering oxygen demand, LVAD devices can also increase myocardial oxygen supply by increasing coronary perfusion pressures. Coronary blood flow occurs in diastole and is a function of the pressure gradient between aortic pressure, *P*_Ao_, and left ventricular diastolic pressure, *P*_LV_, according to the Equation (2):

(2) $$\mathrm{Coronary}\;\mathrm{blood}\;\mathrm{flow}=\frac{P_{Ao_{\mathrm{diastole}}}-P_{LV_{\mathrm{diastole}}}}{\mathrm{Coronary}\;\;\mathrm{artery}\;\mathrm{resistance}}$$

This is known as the diastolic pressure-time index (DPTI) and is shown in Figure 7 together with SPTI (47). The DPTI:SPTI ratio determines the myocardial oxygen supply-demand equation (47) and so an increase in the ratio is associated with improved energy supply. For example, as LVADs displace blood from the LV to the aorta, it increases aortic blood pressure and decreases LV diastolic pressure, resulting in increased diastolic flow according to DPTI and Figure 6 (38, 57). Given that it also lowers the SPTI, due to lower LV pressure (decreased preload), the DPTI:SPTI ratio increases and myocardial oxygen supply- demand mismatch decreases. It is important to note that while the theoretical framework for an improved supply-demand with LVAD may hold true, coronary blood flow also depends on an appropriately opening aortic valve, which can be impaired depending on the orientation of tma-LVADs, while it could also lead to complications such as coronary artery thrombosis (58). Furthermore, studies have shown that as a result of non-pulsatile blood flow, coronary arteries remodel under LVAD support, with increased collagen deposition and breakdown of the internal elastic lamina (59), which can further decrease perfusion to the heart.

### Hemodynamic complications

Despite known improvements in CO and left diastolic pressures after LVAD implantation (60), reported a significant increase in right atrial pressure (RAP) and a significant reduction in pulmonary artery pulsatility index (PAPi) [(systolic pulmonary artery pressure—diastolic pulmonary artery pressure)/central venous pressure] overtime with LVAD implantation. Increased RAP and decreased PAPi indicate progressive worsening of right ventricular (RV) function; a dominant cause of vulnerability in LVAD patients that is linked to poor clinical outcomes (60). PAPi can independently predict patients who will require right ventricular assist device (RVAD) support after LVAD implantation (61, 62). As aforementioned, LVADs shift the LVEDP relationship to the left, causing the pulmonary capillary wedge pressure (PCWP) to decrease, thereby reducing RV afterload (12). However, at higher pump speeds, the increase in RV preload (caused by increased CO) predominates over the reduced RV afterload, and likely contributes to RV dysfunction. Mechanistically, an interventricular septal shift towards the LV occurs, likely causing decreased RV contractility due to abnormal geometry (63). In addition to impaired RV force generation, pre-existing pre-existing disease or transient RV tissue trauma during insertion LVAD may also contribute to development of RV dysfunction (61, 62).

Aortic insufficiency (AI) is another common complication of LVAD, affecting up to 20% of patients within 1 year of implantation (64), and can negatively affect survival (65). To maintain a high net forward blood flow in the presence of AI, LVAD pump speed is often increased in clinical practice (66). However, increasing LVAD speeds also increases regurgitant blood flow, which in turn can contribute to aberrant pulmonary vasculature remodelling and increased RV afterload (66). If the RV intrinsic contractility (*E*_es_) is unable to match the pulmonary vasculature afterload (*E*_a_), RV dysfunction occurs. As aforementioned, the increased preload with increased pump speeds can further contribute to this. Alternative treatment strategies, such as blood pressure control with or without increased pump speed and stratified by patient specific hemodynamic parameters, such as decreased coupling ratio (RV *E*_es_/*E_a_*) (66) or increased pre-operative right-to-left end-diastolic diameter, have been proposed (67).

### Reverse remodelling following LVAD insertion

Mechanical unloading is associated with recovery of cardiac function, in a process known as reverse remodelling (68, 69). We discuss the structural, functional, cellular, and molecular changes that may be associated with this.

### Structural changes

**Figure 8:**
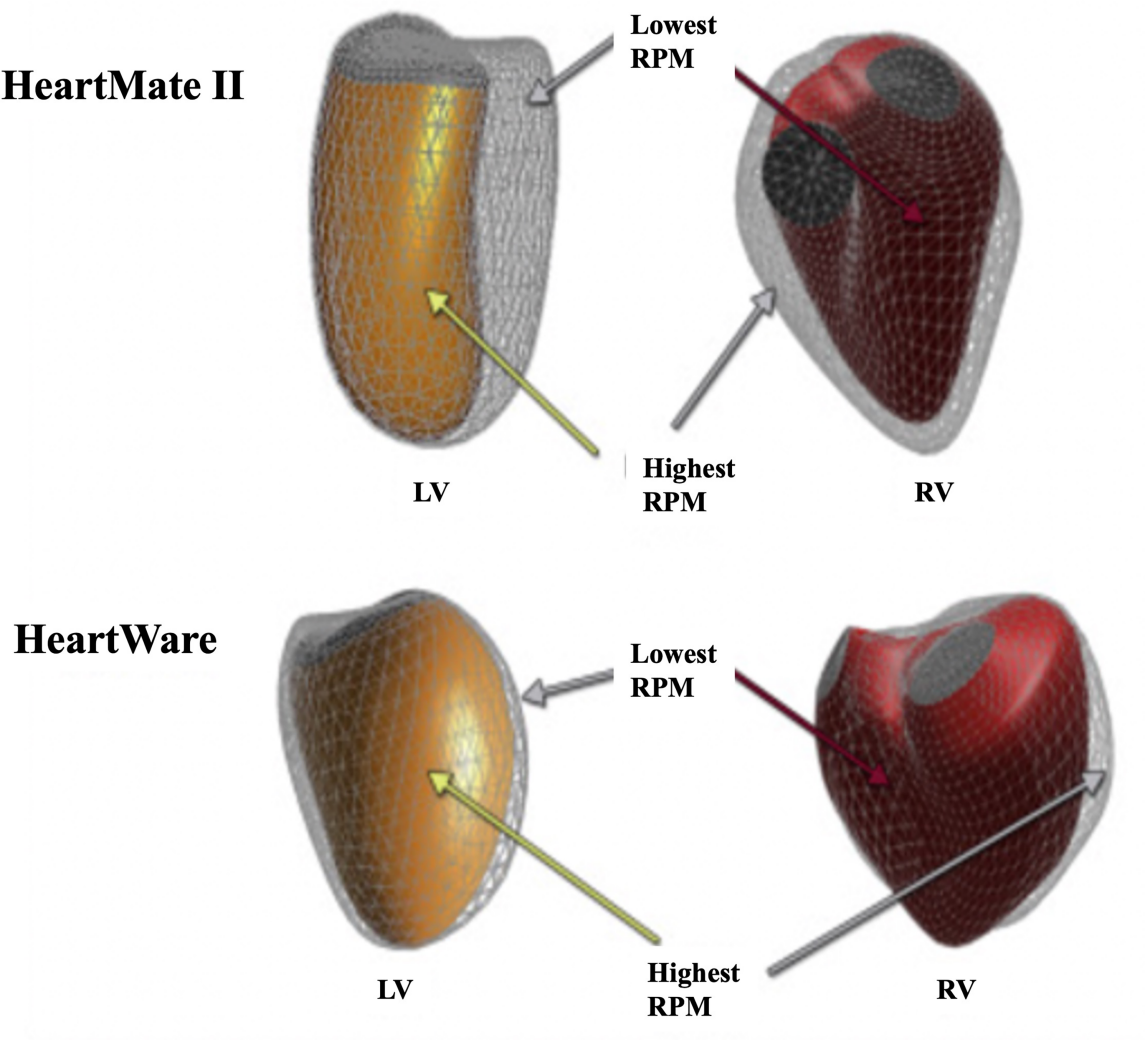
Flow and device specific structural remodelling. 3D illustration of RV (red) and LV (orange) endocardial surfaces geometry obtained following implantation of HeartMate 2 or HeartWare LVADs. Figure adapted from (63).

Long-term LVAD use leads to reduced LV and left atrial (LA) volumes (70, 71). Ventricular geometry is designed for minimal energy expenditure and maximal economy of circulation. Tubular ventricles allow maximal conversion of systolic muscle contraction into required pressure, whereas a remodelled, failing heart adopts a spherical configuration (72). Interestingly, LVAD-mediated changes of the LV's 3D geometry occur immediately upon unloading and are flow-type dependent. For example, intra-abdominal axial-flow LVADs, such with the Heartmate 2, lead to a significantly decreased LV volume and more conical (less spherical) cavity (Figure 8A) (63). Conversely, centrifugal-flow LVADs, such as seen with HeartWare, reduce LV volumes, albeit less, and do not have significant changes in sphericity or conicity (Figure 8B) (63). Device location may explain this distortion, as the inflow LV apex cannula is located sub-diaphragmatically in the intra-abdominal device and thus pulls the apex inferiorly as shown in Figure 3C (63). Interestingly, regardless of the apparent differences in shape, the hemodynamic modifications are similar with both devices, suggesting equivalent degrees of unloading (73). These immediate effects on heart structure may be of clinical relevance when selecting optimal device for each patient (73).

Recently, such changes in ventricular structure have been shown to be maintained with more prolonged device implantation (74). In patients implanted with either HeartMate 2, 3, or HeartWare, and followed before, at 1-month, and 2-months post- implantation the LV shows evidence of reverse structural remodelling as measured by decreased LV end-diastolic volume, decreased LV sphericity and increased conicity (74). However, these findings are not mirrored in the RV, which instead shows increased longitudinal strain suggesting worsening RV function, in line with this being a common complication of LVAD implantation (74).

### Cellular and molecular changes

**Figure 9:**
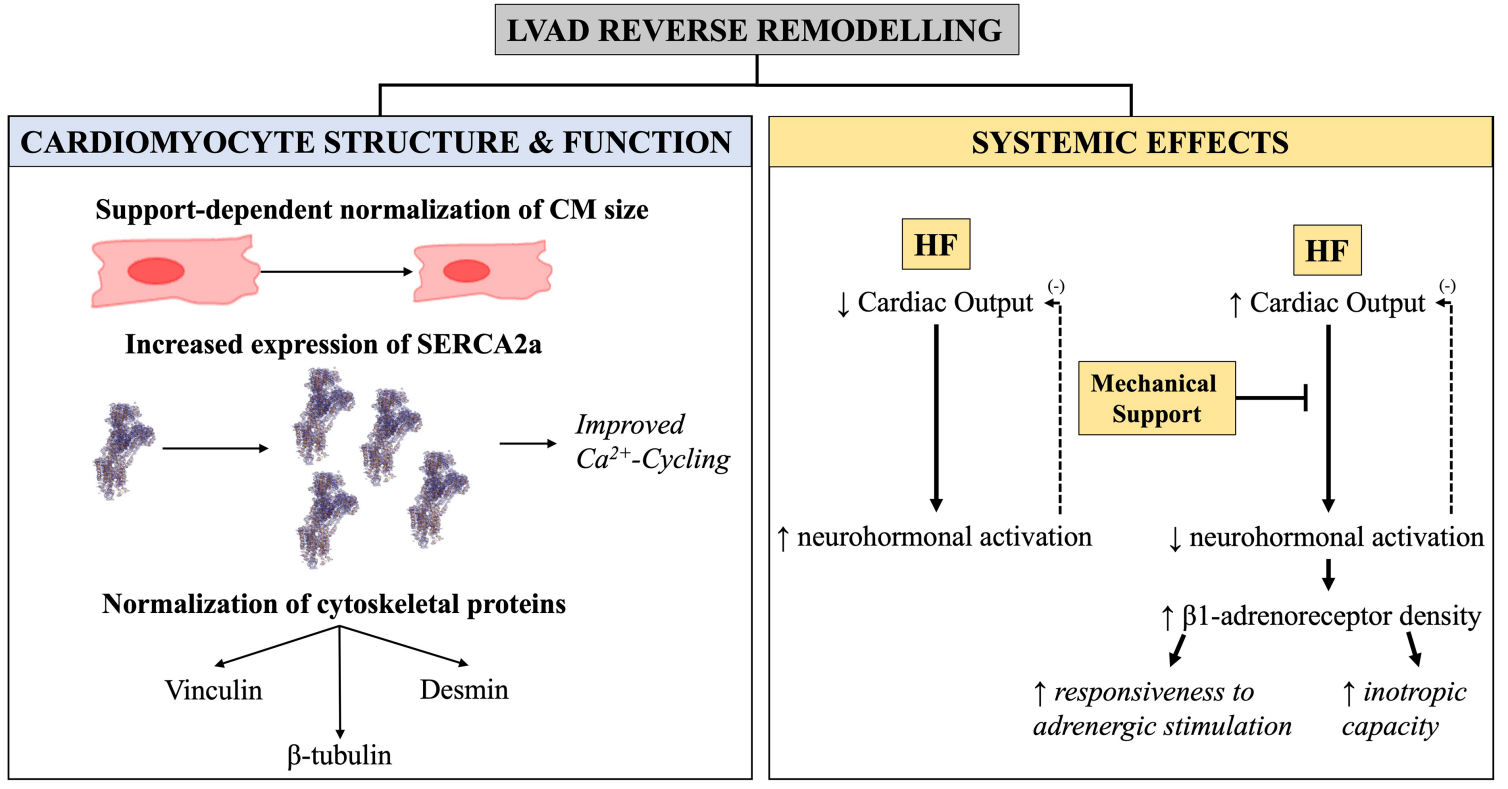
Graphical illustration of the cellular and molecular changes seen with LVAD-induced reverse remodelling. Cellular and molecular changes in LVAD-induced reverse remodelling. (Left) Changes in cardiomyocyte size, expression of Ca^2+^-handling proteins, and cytoskeletal components as a function of mechanical unloading. (Right) Supplementation of cardiac output from mechanical device halts the negative feedback loop [demarcated with (−) here] leading to decreased neurohormonal activation and improved biochemical milieu—these are systemic effects and impact both the RV and LV.

Reverse remodelling from mechanical unloading can be studied at the cellular and molecular level (Figure 9). Cardiomyocyte hypertrophy occurs as a compensatory mechanism to persistent volume- or pressure-overload and is associated with decompensation of cardiac function and progression into heart failure (75). Reduced cardiomyocyte hypertrophy occurs in response to mechanical unloading (43, 76–78), with some studies on pulsatile devices reporting reduction to the point of cardiomyocyte atrophy and degeneration (76, 79, 80), though others have challenged this (81). Mechanical unloading is also associated with decreased myocyte damage (71, 82), and normalisation of the expression of cytoskeletal proteins including vinculin, desmin, ß-tubulin as well as sarcomeric proteins (Figure 9) (83–86). At the intracellular level, prolonged mechanical support normalises expression of genes involved in calcium handling, such as SERCA2a (87), resulting in increased sarcoplasmic reticulum Ca^2+^ content (Figure 9). Increased calcium cycling is associated with recovery of contractile function (13), yet these features are only apparent in the LV.

Of importance is that LVAD-induced reverse remodelling is thought to consist of two arms: those due to the mechanical unloading itself, and those caused by the circulating factors that normalise as a result of supplementation of CO (78, 88). Although the mechanics of the RV are affected by LVAD, with changes in both preload and afterload (89), mechanical unloading impact the LV more than the RV and so are likely to be reflected in the LV more. Conversely, when reversal of phenotype is seen in both RV and LV, then this is likely to be driven by normalisation of the adrenergic axis neurohormonal milieu underlined by supplementation of CO from the device (78). For example, both ventricles exhibit recovery of β-adrenergic responsiveness and receptor density with LVAD support (87, 88, 90, 91). Functionally, this restores myocardial response to ionotropic stimulation by the sympathetic nervous system during exercise, which is the most important regulatory mechanism of cardiovascular performance (Figure 9). Similarly, reversal of ryanodine receptor 2 (RyR2) hyperphosphorylation has been found in both ventricles (88) following LVAD support.

The importance of a correction of the adrenergic system cannot be understated, as the ß-adrenergic system has been repeatedly linked to HF pathogenesis. Down regulation of β1-receptor subtype leads to reduced exercise tolerance and LV inotropic reserve (92), and high levels of sympathetic activity are inversely correlated with prognosis in patients with HF (93).

Extracellular matrix (ECM) composition, structure and its cellular interactions are vital in adaptation to hemodynamic and neurohormonal stressors (94). LVAD-induced changes to collagen, the main structural support molecule of the ECM, is an area of debate. Several studies report reduced myocardial collagen (76, 95, 96), however the majority report no changes (76, 97, 98), or a significant increase (71, 88, 99–101). Increased cross-linked collagen following LVAD support is associated with increased myocardial stiffness (88), so mechanical unloading may lead to decreased compliance. However, such data would not be aligned with the leftward shift in EDPVR seen with LVAD support.

### Combination therapy

Numerous clinical studies demonstrate that neurohormonal blockers reverse pathological cardiac remodelling by restoring normal heart geometry, reducing LV volume and mass, and improving LVEF (102). During LVAD support, concomitant administration of pharmacotherapy (ACE-inhibitors, β-blockers, mineralocorticoids antagonists) enhances the degree of reverse remodelling (103). As aforementioned (31), demonstrated that aggressive pharmacological therapy coupled with mechanical unloading can reverse severe HF in a large proportion of patients with non-ischemic cardiomyopathy. Beside the effects caused by direct mechanical unloading, pump support allows the very high doses required for medication-induced reverse remodelling, whilst maintaining CO and renal perfusion that would otherwise be severely impaired. Schnettler et al. (104) have shown that conventional pharmacological agents (e.g., β-blockers, ACE-inhibitors, mineralocorticoid receptor antagonists) are safe to use in patients with HF on LVAD, yet survival benefit is not different after 1-year compared to those without pharmacological support. Despite clear added effects on reverse remodelling when taken together, optimal combination therapy is yet to be elucidated.

## Conclusion

An understanding of the effects on mechanical support devices on hemodynamics is essential to appreciate their mechanism of action and therapeutic potential. In this review, we have discussed the use of LVADs as mechanical support devices. We have covered their immediate hemodynamic consequences, the long-term effects of these on the mechanical and neurohormonal axis, as well as the key mechanisms of reverse remodelling as well as the phenotypic alterations.
